# Transient anhedonia phenotype and altered circadian timing of behaviour during night-time dim light exposure in *Per3*^−/−^ mice, but not wildtype mice

**DOI:** 10.1038/srep40399

**Published:** 2017-01-10

**Authors:** Bruno Jacson Martynhak, Alexandra L. Hogben, Panos Zanos, Polymnia Georgiou, Roberto Andreatini, Ian Kitchen, Simon N. Archer, Malcolm von Schantz, Alexis Bailey, Daan R. van der Veen

**Affiliations:** 1 Faculty of Health and Medical Sciences, University of Surrey, Guildford, Surrey, UK; 2 Department of Physiology, Federal University of Paraná, Curitiba, Paraná, Brazil; 3 Department of Pharmacology, Federal University of Paraná, Curitiba, Paraná, Brazil; 4 Institute of Medical and Biomedical Education, St George’s University of London, UK

## Abstract

Industrialisation greatly increased human night-time exposure to artificial light, which in animal models is a known cause of depressive phenotypes. Whilst many of these phenotypes are ‘direct’ effects of light on affect, an ‘indirect’ pathway via altered sleep-wake timing has been suggested. We have previously shown that the *Period3* gene, which forms part of the biological clock, is associated with altered sleep-wake patterns in response to light. Here, we show that both wild-type and *Per3*^*−/−*^ mice showed elevated levels of circulating corticosterone and increased hippocampal *Bdnf* expression after 3 weeks of exposure to dim light at night, but only mice deficient for the PERIOD3 protein (*Per3*^*−/−*^) exhibited a transient anhedonia-like phenotype, observed as reduced sucrose preference, in weeks 2–3 of dim light at night, whereas WT mice did not. *Per3*^*−/−*^ mice also exhibited a significantly smaller delay in behavioural timing than WT mice during weeks 1, 2 and 4 of dim light at night exposure. When treated with imipramine, neither *Per3*^*−/−*^ nor WT mice exhibited an anhedonia-like phenotype, and neither genotypes exhibited a delay in behavioural timing in responses to dLAN. While the association between both *Per3*^*−/−*^ phenotypes remains unclear, both are alleviated by imipramine treatment during dim night-time light.

Night-time exposure to light has increased as a consequence of the use of electrical illumination in industrialised societies^1^,^2^. This mistimed light exposure is associated with alterations in mood and affect, and is accompanied by changes in sleep and circadian rhythms^3^. Sleep-wake timing is driven by timing of internal circadian clocks, and at the same time is also directly altered by light exposure. It remains unclear whether the contribution of changes in circadian clocks to the aetiology of mood disorders is only negative, or whether changes in sleep-wake timing are also a positive response, aimed at sheltering from activity-related light exposure at times when the circadian system is most sensitive to light^4^.

Changes in mood and depression are often associated with circadian rhythm disturbances such as dampening^5^ or loss^6^ of core body temperature rhythms, arguing for an indirect aetiology of mood disorders via circadian timekeeping mechanisms. A striking example of this indirect effect of light on mood is seasonal affective disorder, which is characterised by altered circadian timing and associated depressive episodes during the short photoperiods of winter and recovery of affect under long photoperiods in the summer^7^,^8^. However, the mechanisms involved with altered circadian timing in the pathophysiology of mood disorders are unclear, and it remains possible that light-induced changes in mood occur independently of the changes in circadian timing.

Light is perceived through rod and cone photoreceptor pathways, as well as through the more recently identified non-visual pathway involving intrinsically photosensitive retinal ganglion cells (ipRGC), expressing melanopsin^9^. In the last few years, a direct, ipRGC-dependent, pathway has been demonstrated, whereby light can cause mood and cognitive disruption without changes in sleep or circadian timing^10^. Indeed, mice that lack the melanopsin-expressing ipRGCs (*Opn4*^*aDTA/aDTA*^) do not exhibit impaired mood during exposure to a short 7-h light:dark cycle (3.5:3.5 h), despite them having intact image forming pathways^11^. These findings suggest that the often-observed changes in circadian timing system caused by mistimed light exposure may not be directly causal to the changes in affect.

In animal models of depression caused by mistimed light exposure, both constant darkness^12^ and constant light^13^,^14^,^15^ have been shown to increase immobility in the forced swim test and reduce sucrose preference, both of which are measures of depressive-like behaviour. Moreover, neonatal exposure to constant light prevents circadian arrhythmicity and depressive-like behaviour induced by constant light exposure in subsequent adulthood^13^,^16^,^17^, further suggesting an indirect role of light through the disruption of the circadian rhythms. Depressive-like behaviour induced by constant light was partially rescued when mice were given the opportunity to shelter from light in an opaque tube^14^, which suggests a role for the direct effect of the light in the development of mood. However informative, these experiments involving constant light conditions lead to loss of light-dark synchronised circadian cycles, and do not model the altered circadian timing observed in the human condition.

More recently, naturalistic approaches have been used such as exposure to short photoperiods^18^ and, in particular, exposure to dim light during the dark phase^19^. A main advantage of this “dim light at night” (dLAN) approach is that, under these conditions, animals remain synchronised to a 24-hour cycle, allowing the investigation of any putative changes in diel timing relative to an external light-dark cycle. Prolonged exposure to dLAN promotes depressive-like behaviour in various species^20^,^21^,^22^ and associates with neurobiological alterations such as increased hippocampal TNF-α^20^, decreased hippocampal BNDF levels^21^, and decreased hippocampal CA1 dendritic arborisation^19^.

In order to understand what role light-associated changes in circadian timing play in the onset of depressive-like behaviour in dLAN, we turned to the *Per3*-deficient (*Per3*^*−/−*^) mouse model. Mice lacking PERIOD3 are synchronised to light:dark cycles in a normal way, and exhibit normal immediate responses to light-induced phase shifts^23^. While these aspects of the circadian system are thus intact in *Per3*^*−/−*^ mice, they exhibit reduced behavioural circadian responses to constant light, and show less negative masking of behaviour by light (i.e., less activity inhibition by light)^24^,^25^. Because of this previously described phenotype, in which *Per3*^*−/−*^ mice exhibit an attenuated circadian response to prolonged light exposure, this rodent model can be used to investigate the development of mood disorders mediated by light at night, owing to the increased direct changes in behavioural timing in response to light, rather than indirect effects via the circadian system in the mice.

We hypothesise that if the circadian system is part of the pathophysiology of mood disorders in dLAN, *Per3*^*−/−*^ mice would exhibit an altered time of onset of anhedonia-like behaviour than wild-type (WT) mice do. We observed that *Per3*^*−/−*^ mice showed both a reduced delay in circadian timing of behaviour in weeks 1, 2 and 4 of night-time dim light exposure, and a transient reduction of sucrose preference in weeks 2 and 3 of night-time dim light exposure. The anhedonia-like phenotype as measured in our sucrose preference test is associated with a depressive-like phenotype^26^. The nature of the association between these changes in behaviour and anhedonia-like responses in *Per3*^*−/−*^ mice remains unclear, but imipramine treatment alleviates both phenotypes of *Per3*^*−/−*^ mice in response to dim light at night.

## Results

### Baseline phenotype in the Forced Swim test

**Figure 1:**
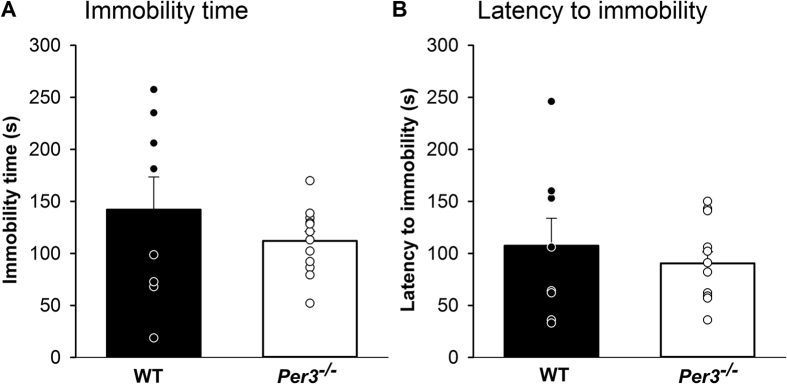
Immobility time (**A**) and latency to immobility (**B**) in the Forced Swim test in WT and *Per3*^*−/−*^ mice. Data is shown as histograms of means and standard error of the mean, circles indicate individual values. n = 8–12 males per group.

Neither total immobility (Fig. 1A) nor latency to the first immobility (Fig. 1B) were significantly different between the genotypes (t = 1.09, p > 0.05) and (t = 0.67, p > 0.05).

### Sucrose preference

**Figure 2:**
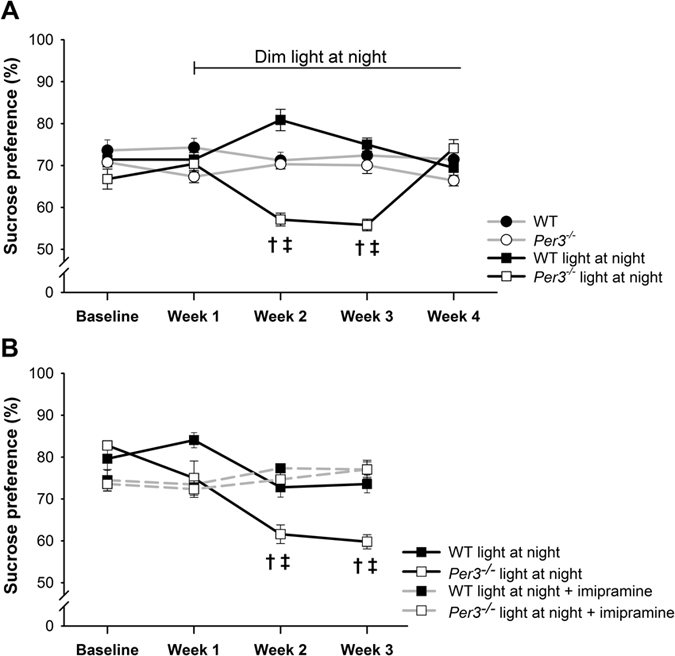
Sucrose preference in WT and *Per3*^*−/−*^ mice in (**A**) LD or dim light at night and (**B**) dim light at night, treated with tap water or imipramine in the drinking water. Values are presented as means and standard error of the mean. ^†^p < 0.05 in comparison with the control group. ^‡^p < 0.05 in comparison with baseline. n = 6–8 males per group.

At the end of the second week of dLAN, *Per3*^*−/−*^ mice, but not wild type mice, exhibited a significantly reduced sucrose preference. Figure 2A shows that this reduced sucrose preference remained significantly reduced during week 3, and then returned to the same levels as the WT mice (genotype: F_1,27_ = 27.90, p < 0.001; light schedule: F_1,27_ = 1.42, p > 0.2; genotype x light condition: F_1,27_ = 5.19; p < 0.05; genotype x light schedule x week: F_4,108_ = 18.3, p < 0.001).

In a second experiment, we confirmed a reduction of sucrose preference in *Per3*^*−/−*^, which was not observed in wild type mice, in week 2 and 3 after the start of dLAN. Treatment with the tricyclic antidepressant imipramine prevented the development of reduced sucrose preference in the *Per3*^*−/−*^ mice (Fig. 2B, genotype: F_1,26_ = 10.0, p < 0.01; treatment: F_1,26_ = 0.9, p > 0.2; genotype x treatment: F_1,26_ = 5.5, p < 0.05; genotype x treatment x week: F_3,78_ = 4.2, p < 0.01). *Post hoc* analysis showed that sucrose preference was significantly reduced during weeks 2 and 3 of dLAN exposure in *Per3*^*−/−*^ mice that received no imipramine treatment (p < 0.001), but not in imipramine-treated *Per3*^*−/−*^mice (Fig. 2B).

### Timing of behavioural activity

During light-dark entrained conditions, there was no difference in the timing of behavioural activity of WT and *Per3*^*−/−*^ mice, as measured by the timing of the first and second peak of nocturnal behavioural activity, but a timing difference between genotypes was observed when the mice were exposed to dLAN (PROC MIXED, genotype: F_1,233_ = 4.59; dLAN: F_1,233_ = 25.75; treatment: F_1,233_ = 6.34; genotype x dLAN: F_1,233_ = 10.47; genotype x treatment: F_1,233_ = 5.83; p < 0.05). Supplemental Figure 1 shows average activity profiles and respective average Fourier fits for each week during baseline, dLAN and dLAN with imipramine conditions for both wild-type and *Per3*^*−/−*^mice. Figure S2 shows example actogram of *WT and Per3*^*−/−*^going through a dim light at night protocol without, and with Imipramine.

**Figure 3:**
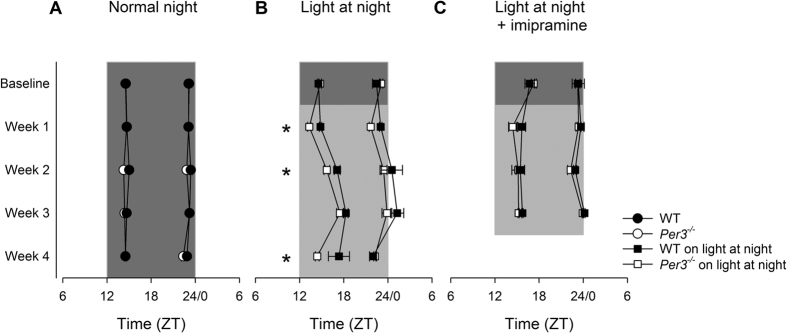
Phase of activity of the first and second peaks of activity. (**A**) LD. (**B**) dLAN (**C**) dLAN and imipramine treatment. *p < 0.05 in comparison with WT. Circles represent mean and standard error of the mean of the first and second peaks of activity, areas indicate time between both peaks. Grey areas: dim light at night, dark grey areas: dark phase of the cycle. n = 6–8 males per group.

Figure 3A shows that both WT and *Per3*^*−/−*^mice exhibit stable and invariant timing of behavioural activity peaks in light-dark entrained conditions (LSMEANS p > 0.05). Figure 3B shows a similar entrainment patterns in the baseline week, but during the exposure to dLAN, the *Per3*^*−/−*^ mice showed an attenuated delay in timing of the first behavioural activity peak (LSMEANS p < 0.05 in weeks 1, 2 and 4 of dLAN), indicating that *Per3*^*−/−*^ mice were more active in the early night than WT mice are under these conditions.

Imipramine treatment resulted in similar timing of behavioural activity between WT and *Per3*^*−/−*^mice in both the baseline and dLAN conditions (Fig. 3C, LSMEANS P > 0.05). Importantly, imipramine treatment prevented the dLAN-induced phase delay in week 2 (LSMEANS p < 0.05), whereas imipramine treatment in WT mice prevented the phase delay in weeks 3 and 4 (p < 0.001).

Although not plotted in a single graph, dLAN phase delayed the second peak of activity similarly in *Per3*^*−/−*^ and WT mice (LSMEANS P < 0.0001, Fig. 3B,C), an effect that was rescued by imipramine treatment (LSMEANS p < 0.05).

### Circulating corticosterone levels

**Figure 4:**
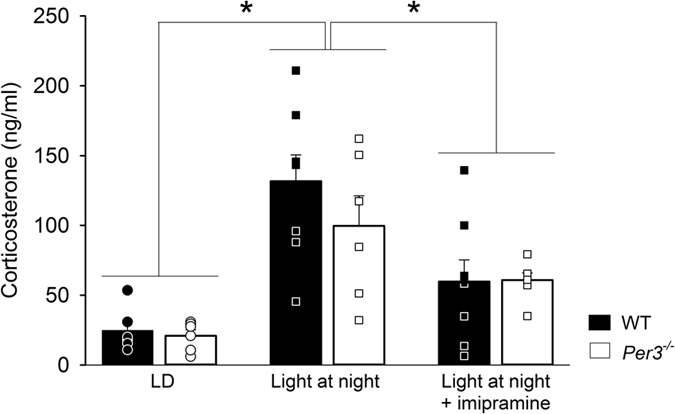
Plasma corticosterone collected at ZT0 (lights on). Mice were killed at the end of week 4 for LD and week 3 for dLAN. Data is shown as histograms of means and standard error of the mean, circles indicate individual values. n = 5–8 males per group. *p < 0.05.

As shown in Fig. 4, plasma corticosterone levels were increased in both genotypes after dLAN exposure with and without imipramine treatment (group: F_2,36_ = 19.72, p < 0.001; genotype: F_1,36_ = 1.35, p > 0.05, genotype x group: F_2,36_ = 0.56; p > 0.05). The level of plasma corticosterone in the imipramine-treated mice was intermediate to that of the light-dark entrained mice and the dLAN treated mice without imipramine (p < 0.05).

### Hippocampal gene expression

**Figure 5:**
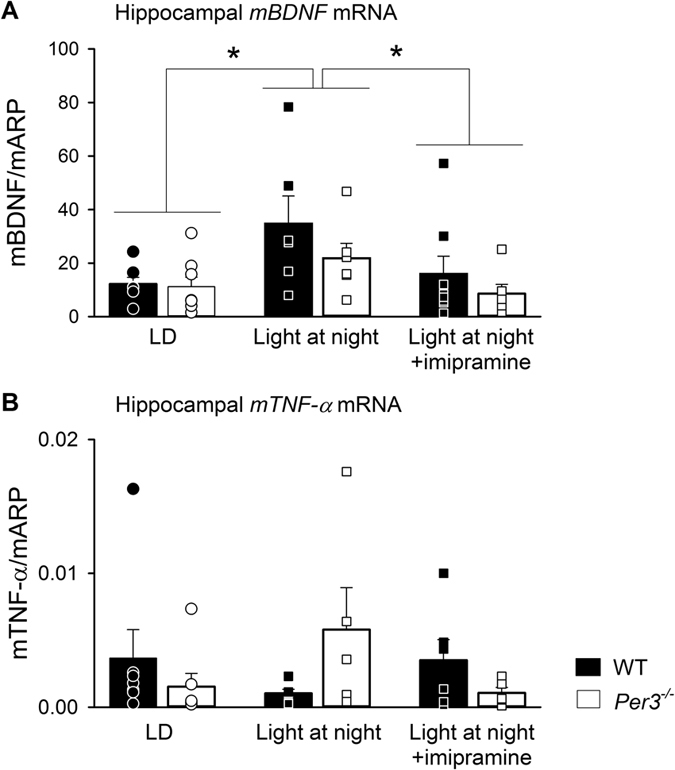
Expression of hippocampal *Bdnf* (**A**) and hippocampal *Tnf-α* (**B**). Data is shown as histograms of means and standard error of the mean, circles indicate individual values. Mice were killed at the end of week 4 for LD and week 3 for dLAN. n = 5-8 males per group. *p < 0.05.

*Bdnf* expression in the hippocampus was increased in both genotypes after dLAN exposure with, and without imipramine treatment (Fig. 5), but the level of hippocampal *Bdnf* expression in the imipramine-treated mice was intermediate to that of the light-dark entrained mice and the dLAN treated mice without imipramine (group: F_2,35_ = 4.32, p < 0.05; genotype: F_1,35_ = 2.23, p > 0.05, genotype x group: F_2,35_ = 0.52; p > 0.05).

For the hippocampal *Tnf-α* expression, a significant interaction between genotype and group was found (F_2,30_ = 3.99, p < 0.05). However, *post hoc* analysis did not reveal any significant contrasts.

## Discussion

We have shown that, after two weeks of exposure to dim light at night, mice deficient in the PERIOD3 protein develop an anhedonic-like phenotype. Both wild type and *Per3*^*−/−*^mice exhibited increased levels of circulating corticosterone and hippocampal *Bdnf* expression in response to dim light at night. The rapid development of a reduced sucrose preference phenotype in *Per3*^*−/−*^ mice in the second week after exposure to dim light at night, was much earlier than normally expected, as it has been well documented that after two weeks of dim light at night, mice normally do not develop a depressive-like phenotype^14^,^19^,^20^,^21^,^27^. The early onset of this transient anhedonia-like phenotype in weeks 2 and 3 of dim light at night, partially overlapped with the significant attenuation in the behavioural phase delay during weeks 1, 3, and 4 of this dim light at night protocol. *Per3*^*−/−*^ mice did not delay their behavioural activity onset as much as wild type mice do during these weeks, which is in line with our previously published observations^24^,^25^. The weeks in which *Per3*^*−/−*^ mice exhibit this significant attenuation in the delay in activity in comparison with WT mice did not match entirely with weeks in which *Per3*^*−/−*^ mice showed reduction in sucrose preference, and we have no evidence for a causal relationship. Interestingly, a recent publication shows that mice carrying a humanised *Per3* transgene similarly exhibit a depressive-like phenotype, as well as changes in the molecular circadian clock^28^.

Anhedonia, defined as a diminished ability to experience pleasure in previously rewarding stimuli, is widely considered as a core symptom of human depression^29^,^30^,^31^,^32^. Importantly, more than half of the individuals suffering from bipolar depression also experience significant levels of anhedonia^33^, and depressed patients with reported anhedonia have a poorer treatment prognosis than their non-anhedonic counterparts^34^,^35^,^36^, highlighting the significant role of anhedonia in these affective disorders. In animal models of depression, hedonic deficits, and more specifically decreased sucrose preference, can be induced by chronic stress^37^,^38^,^39^,^40^, chronic social defeats^41^,^42^ and chronic administration of corticosterone^43^,^44^,^45^. All these models have been shown to induce biochemical changes similar to those observed in depressed patients^46^,^47^,^48^,^49^.

In line with this association of reduced sucrose preference with a depressive-like phenotype, pharmacological treatment with the antidepressant imipramine prevented the development of the anhedonia-like behaviour in *Per3*^*−/−*^ mice, similarly to the reported effect of citalopram in preventing increased immobility in the FST test in WT mice exposed to dLAN^50^. Imipramine treatment also reduced the delay in the circadian activity in both *Per3*^*−/−*^ and WT mice. Whether the imipramine-associated change in the behavioural response of *Per3*^*−/−*^ mice in dLAN is part of the mode of action of this antidepressant is unclear, but imipramine treatment in itself was reported not to change any measure of the circadian rhythms in naïve hamsters^51^, suggesting that it may act upstream of the circadian clock in relation to mood regulation, which has been suggested before, even though ambiguously^51^,^52^,^53^,^54^. It is unclear what from this study whether there are underlying mechanisms which may link timing of behavioural activity in dLAN to reduction in sucrose preference, but it may be hypothesised that delaying the activity bout reduces light exposure during activity in the early night, when the circadian system responds strongly to light^4^,^55^.

In a previous study using wild type mice, dim light at night triggered depressive-like behaviour only after four weeks of night time light exposure^21^, also measured as sucrose anhedonia and the forced swim test. The absence of a sucrose anhedonia in wild type mice during the four weeks of dLAN that we used is therefore expected, and the transient reduction of sucrose preference in our *Per3*^*−/−*^ mice is uncharacteristically rapid. In the fourth week of dLAN, the reduction in sucrose preference in the *Per3*^*−/−*^ mice spontaneously subsided. Whilst some variability in sucrose preference is commonly observed during a chronic mild stress model, including the spontaneous recovery during the chronic stress^56^, it is unclear why the early onset of anhedonia-like phenotype of *Per3*^*−/−*^ mice subsides at in week 4 during dLAN exposure. This spontaneous recovery could be part of the ongoing long-term changes in affect, which also result in the development of depressive-like behaviour in WT rodents only after 4–8 weeks^14^,^19^,^20^,^21^,^27^.

C57bl/6 mice do not produce significant rhythms in melatonin^57^, in contrast to the C3H/HeNHsd mice used in the previous work linking dim light at night to the development of depressive-like behaviour^21^. Therefore, proposed pathways whereby suppression of melatonin production through dim light may add to the anhedonia-like phenotype effects of dLAN are unlikely to be functional in our model, and the results obtained here point to mechanisms independent from melatonin.

Prolonged dLAN exposure increased plasma corticosterone levels and hippocampal *Bdnf* levels in both wild-type and *Per3*^*−/−*^ mice, irrespective of the affective state of the animals, and imipramine treatment attenuated the increase in both. Several stressors, which increase corticosterone, can be used to promote depressive-like behaviour in rodents^39^ and exogenous corticosterone can also lead to depressive-like behaviour^58^. Although chronic stress usually leads to reduction of BDNF^58^, in our study, *Bdnf* expression was increased after dLAN exposure. However, an increase in hippocampal *Bdnf* expression does not necessarily reflect increased activation of its receptor, TrkB. It is known that pro-BDNF, when not converted to BDNF, may actually lead to opposite effects on BDNF, inducing apoptosis instead of cell survival^59^. An increase in pro-BDNF may be what is reflected by the increase in hippocampal *Bdnf* expression after exposure to dLAN. In addition, early life stress increased *Bdnf* expression and neurogenesis in young adults, but decreased in aged rats^60^. One could thus interpret that our changes in *bdnf* expression and circulating corticosterone levels represent early activation of the peripheral stress system, which is a prelude to a depressive-like phenotype that has been reported to occur later^21^, and are unrelated to the early onset sucrose-anhedonia phenotype linked to behavioural timing described here. However, it should be noted that a single time point (ZT0) was used for the analysis, and the possibility of differences at other time points cannot be excluded.

Levels of hippocampal *Tnf-α* expression varied significantly between imipramine treatment and genotypes, and although we may suspect an increase in hippocampal *Tnf-α* expression in *Per3*^*−/−*^ mice that is associated with their anhedonia-like behaviour in dLAN, no significant differences could be identified using *post hoc* tests. Depression has been widely associated with increased inflammatory processes^61^, with anti-inflammatory drugs having antidepressant-like effects in the chronic mild stress model in rats^62^ and being used as an add-on therapy for major depression^63^. As it stands though, our data does not confirm that inflammation plays a particular role in early onset of the sucrose anhedonia induced by dLAN in *Per3*^*−/−*^ mice.

One limitation of this study is that we chose to only pursue sucrose preference for evaluation of the depressive-like behaviour, and discontinued the forced swim test after the baseline measurements, which showed immobility times in line with previously published work^64^,^65^. The sucrose preference test is less stressful than other frequently used tests, such as forced swim test and tail suspension test, and is more appropriate for repeated measurements in chronic studies. Moreover, the ‘acute’ nature of the forced swim test and tail suspension test would only allow us a measurement at a single circadian time point, and due to the stress associated with these tests may even act as a circadian timing signal which would confound our current approach. The sucrose preference test is related to anhedonia, which is a core symptom of major depression^26^, and while the forced swim test is most frequently used to screen putative antidepressant-like drugs, we really do not know what it measures: despair, hopeless or an adaptive behaviour for energy conservation^66^.

We have shown that *Per3*^*−/−*^ mice rapidly develop a transient anhedonic-like phenotype, as well as an attenuated delay in the sleep-wake timing during dim light at night as compared to wild-type mice. This early onset of reduced sucrose preference in *Per3*^*−/−*^ mice was prevented by imipramine treatment, which also prevented the delay in behavioural activity under dLAN in both wild type and *Per3*^*−/−*^ mice. These *Per3*^*−/−*^ specific changes in measures associated with affect appear unrelated to the general levels of stress caused by dim light at night, as measured by hippocampal *Bdnf* expression and circulation corticosterone in both genotypes. While the association between the anhedonia-like phenotype and changes in behavioural timing remains unclear, both *Per3*^*−/−*^ phenotypes are alleviated by imipramine treatment during dim night-time light.

## Materials and Methods

### Ethics statement

All mouse experimental procedures were approved by the U.K. Home Office. All mouse experiments were carried out in accordance with the guidelines and regulations of the U.K. Home Office.

### Subjects and housing

All mice were backcrossed to a C57BL/6J genetic background for at least 10 generations. Mice homozygous for a targeted disruption of the *Per3* gene^23^, and their wild-type controls were bred in-house from heterozygous breeding pairs and genotyped as published elsewhere^24^. All experiments were performed with male mice. Food and water were available *ad libitum*. Individual cage illumination was supplied by LEDs (NSPW500BS, Nichia Europe BV, Amsterdam, the Netherlands) through frosted glass. Light intensity was measured with the light-sensor at the cage bottom directed toward the light.

For the Forced Swim test, mice were housed in groups of 2–5 per cage in a temperature-controlled environment with a 12:12-h light/dark cycle (150 lux in light phase, light on at: 07:00 h GMT).

For dLAN experiment, adult (8 weeks old) mice were individually housed in light-tight, sound-attenuated cabinets. Home cage locomotor activity was continuously recorded through passive infrared detectors (Gardtec Gardscan MX PIR, Gardiner Technology, Rochdale, UK) connected to a PC (ClockLab, Actimetrics, Wilmette, IL), and analysed in 1-minute bins. Animals were kept in 12:12-h light/dark cycle (150 lux for light phase, lights on: 10:00 h).

### Forced Swim test

To evaluate whether *Per3*-deficient mice had a different anhedonia-like phenotype than WT mice under baseline conditions, the Forced Swim test was performed as previously published^67^. Briefly, animals were manually handled for habituation for three consecutive days previous to testing between ZT2 and ZT6. On the testing day, animals were placed in a transparent cylinder of water at 23–24 °C for 6 min with environmental light levels of 40 lux, again at ZT2-ZT6 Total immobility time, and latency to the first immobility episode were scored as measures of depressive-like behaviour.

### Dim light at night (dLAN)

After two weeks of acclimation under 12:12-h light/dark cycle (150 lux in light phase, lights on at 10:00 h) and single housing, a baseline sucrose preference test (described below) was performed and mice were randomly assigned to remain in either a LD cycle, or assigned to the dim light at night cycle (150 lux in light phase, 5 lux in dim light phase, brighter lights on at 10:00 h, 7–8 mice per group)^21^. Sucrose preference tests were performed weekly to evaluate the time-course for the development of any depressive-like behaviour. After four weeks mice from the LD group were killed at ZT0, and trunk blood was collected in heparin-coated tubes for corticosterone measurements, and brains were collected in RNAlater (Sigma Aldrich, St Louis, MO) and frozen at −80 °C for subsequent dissection and qPCR. Since there was spontaneous recovery from the reduction in sucrose preference, only the control (LD) mice had their tissue collected in this experiment.

### dLAN with imipramine treatment

In order to investigate whether the dLAN-induced depressive-like behaviour was rescued trough antidepressant treatment, a second experiment was performed similarly to the first one. In this experiment, following two weeks of 12:12-h light/dark cycle, mice were assigned to a control group (dLAN only), or the imipramine treatment group (6–8 mice per group). All mice were exposed to the dLAN cycle and killed at the end of week 3 (at this time there was a significant reduction in sucrose preference in the *Per3*^*−/−*^ mice under dLAN without imipramine treatment), and blood and tissues were collected for analysis. Imipramine pre-treatment started two weeks before the onset of dLAN and continued throughout the experiment in order to prevent the dLAN-induced reduction in sucrose preference [it has been reported that 7 days of imipramine pre-treatment through drinking water could prevent stress-induced anhedonia^68^].

In order to minimise disturbances to the animal, imipramine was offered through the drinking water at a concentration of 128 mg/L. Based on a body mass of ~30 g, and a consumption of ~3.5 mL of water per day, this would result in a dose of 10–15 mg/kg imipramine per day. This schedule of treatment was previously used by members of our group with the same target dose in rats^13^

### Sucrose preference test

The sucrose preference test was performed as previously published^39^,^69^, with minor modifications. Mice were offered free choice of two drinking bottles, one containing tap water, and the other containing a 2% sucrose solution in tap water. After 24 hours (from ZT23 to ZT23, to avoid unneeded arousal due to bottle exchange), consumption from both bottles was measured, and mice were restored to a single bottle containing tap water. Sucrose preference is always shown as percentage of sucrose solution intake in relation to total liquid intake. Mice were first habituated to the sucrose 7 days before the start of the dLAN schedule, by offering solely the sucrose solutions for 24 hours, followed by 48 hours of free choice between tap water and sucrose solution. The sucrose preference tests were conducted on the last day of each of the experiment, thus day 0, 7, 14, 21 and 28, corresponding to the last day of baseline in LD, week 1, week 2, week 3 and week 4 in dLAN.

### Gene expression using quantitative PCR

The hippocampus and a block of prefrontal cortex were manually dissected from the brains, which had been stabilised in RNA Later (Invitrogen, CA, USA) upon collection. mRNA was extracted using the RNeasy Mini Kit (Qiagen, Hilden, Germany) according to the manufacturer’s instructions. For each sample, both the level of expression of the gene of interest and housekeeping gene (*Arp*) was performed using Taqman qPCR. The primers and probe for each gene are shown in Table 1.

### Corticosterone measurement

1–1.5 mL of trunk blood from each animal was collected in heparin-coated tubes, and immediately placed on ice. Samples were centrifuged at 4 °C (>16 000 RCF) and plasma was stored at −20 °C until processing. Corticosterone levels were assessed using a rat/mouse corticosterone [^125^I] radioimmunoassay kit (MP Biomedicals, New York, NY), according to the manufacturer’s instructions. The corticosterone levels were determined in technical duplicates, and all samples were assessed in a single assay.

### Statistics

The results of the Forced Swim test were analysed using unpaired, two-tailed t-tests. Sucrose preference, corticosterone and gene expression levels were statistically tested using by 3-way ANOVA (with repeated measures where appropriate), with factors genotype, light condition and imipramine treatment, and individual as repeated measures. Where ANOVA tests indicated statistical significant effects, *post hoc* pairwise comparisons were made using the Newman-Keuls test. All tests were performed in Sigmaplot version 12.3 (Systat Software Inc, San Jose, CA).

Determination of circadian timing of behavioural activity started with the identification of the first and second peak phases of locomotor activity by means of fitting two harmonic Fourier curves through the Z-scored behavioural data of the last two days of each week, for each mouse for each week. On some occasions, no first or second peak could be fitted due to low activity, resulting in 38 out of a potential total of 275 peaks being omitted from the analysis, which is a loss of less than 10%. The two separate groups of animals assigned to dLAN at different occasions (in the first and second experiment) did not show significant differences in any of the measures, and are therefore represented as a single group to increase statistical power. All statistics were performed using procedure MIXED in SAS 9.2 (SAS Institute, Cary, NC), and where statistical significant effects were identified, the post-hoc comparison was based on least square means (LSMEANS).

Results are expressed as mean ± Standard Error of the Mean (SEM).

## Additional Information

**How to cite this article**: Martynhak, B. J. *et al*. Transient anhedonia phenotype and altered circadian timing of behaviour during night-time dim light exposure in *Per3*^−/−^ mice, but not wildtype mice. *Sci. Rep.* **7**, 40399; doi: 10.1038/srep40399 (2017).

**Publisher's note:** Springer Nature remains neutral with regard to jurisdictional claims in published maps and institutional affiliations.

## Supplementary Material

Supplemental Figures

## Figures and Tables

**Table 1:** List of primers and probes.

Target	Forward	Reverse	Probe
*Tnf-α*	TACTTAGACTTTGCGGAG	AGAGTAAAGGGGTCAGAG	AGGTCTACTTTGGAGTCATTGCTC
*Bdnf*	GGGTCACAGCGGCAGATAAA	GCCTTTGGATACCGGGACTT	TCTGGCGGGACGGTCACAGTCCTA
*Arp*	GGGATTCGGTCTCTTCGACTAA	GCCTTTATTTCCATCTTTCTCAAATT	CCCGCCAAAGCAACCAAGTCAGC
