# Supplementary material for: Transient anhedonia phenotype and altered circadian timing of behaviour during night-time dim light exposure in Per3−/− mice, but not wildtype mice
Source: Sci Rep. 2017 Jan 10;7:40399. doi: 10.1038/srep40399 (PMC5223197; doi:10.1038/srep40399)

Supplemental information for:

Transient anhedonia phenotype and altered circadian timing of behaviour during night-time dim light exposure in *Per3*$^{-/-}$ mice, but not wildtype mice.

Bruno Jacson Martynhak PhD[1, 2], Alexandra L. Hogben PhD[1], Panos Zanos PhD[1], Polymnia Georgiou PhD[1], Roberto Andreatini PhD[3], Ian Kitchen PhD[1], Simon N Archer PhD[1], Malcolm von Schantz PhD[1], Alexis Bailey PhD[1,4], Daan R van der Veen PhD[1]

[1] Faculty of Health and Medical Sciences, University of Surrey, Guildford, Surrey, UK

[2] Department of Physiology, Federal University of Paraná, Curitiba, Paraná, Brazil

[3] Department of Pharmacology, Federal University of Paraná, Curitiba, Paraná, Brazil

[4] Institute of Medical and Biomedical Education, St George's University of London, UK

Supplemental Figure S1:

Group average of wild-type and *Per3*$^{-/-}$ mouse overall activity profiles during the last 2 days of each week of baseline and subsequent dim light at night conditions for untreated (A) and Imipramine treated (B) mice. Curves represent the Fourier fits through the group average profiles, and triangles indicated the group averages of the first and second peak phase of individual Fourier fits.

Figure on next page

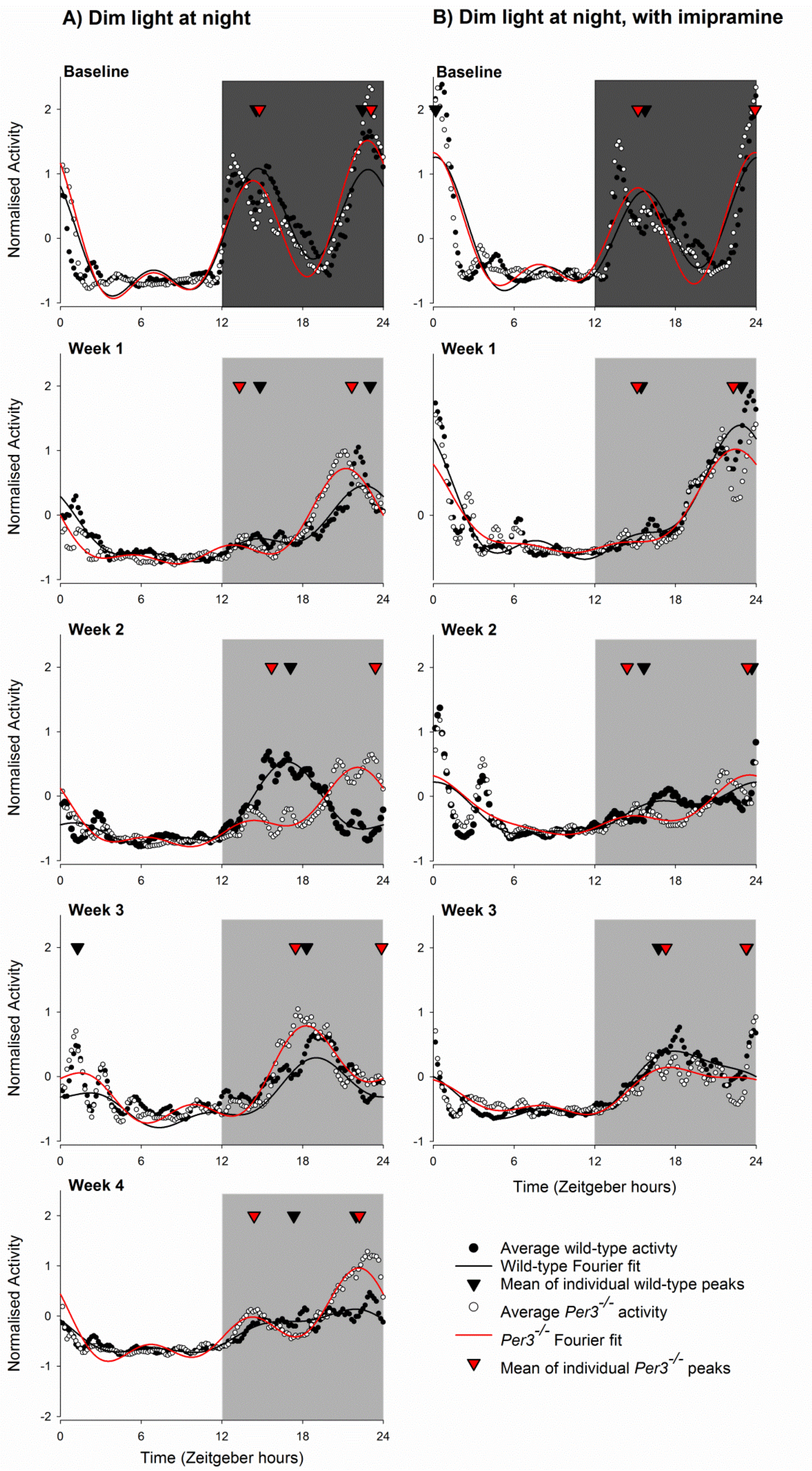
A) Dim light at night
B) Dim light at night, with imipramine
Baseline
Week 1
Week 2
Week 3
Week 4
Normalised Activity
Time (Zeitgeber hours)
Average wild-type activty
Wild-type Fourier fit
Mean of individual wild-type peaks
Average Per3-/- activity
Per3-/- Fourier fit
Mean of individual Per3-/- peaks

Supplemental Figure S2:

Double plotted representative actograms of untreated wild-type (top left) and *Per3*$^{-/-}$ (top right) mice, and Imipramine treated wild-type (bottom left) and *Per3*$^{-/-}$ (bottom right) mice subjected to 10 days of 12 hours light, 12 hour dark conditions, followed by dim light at night. Dark grey areas indicates complete darkness, light grey areas indicate dim light at night (5 lux), white areas indicate light during the day (150 lux). Time of day on the x-axis is indicated in Zeitgeber hours, where Zeitgeber time 0 is the time of lights on.

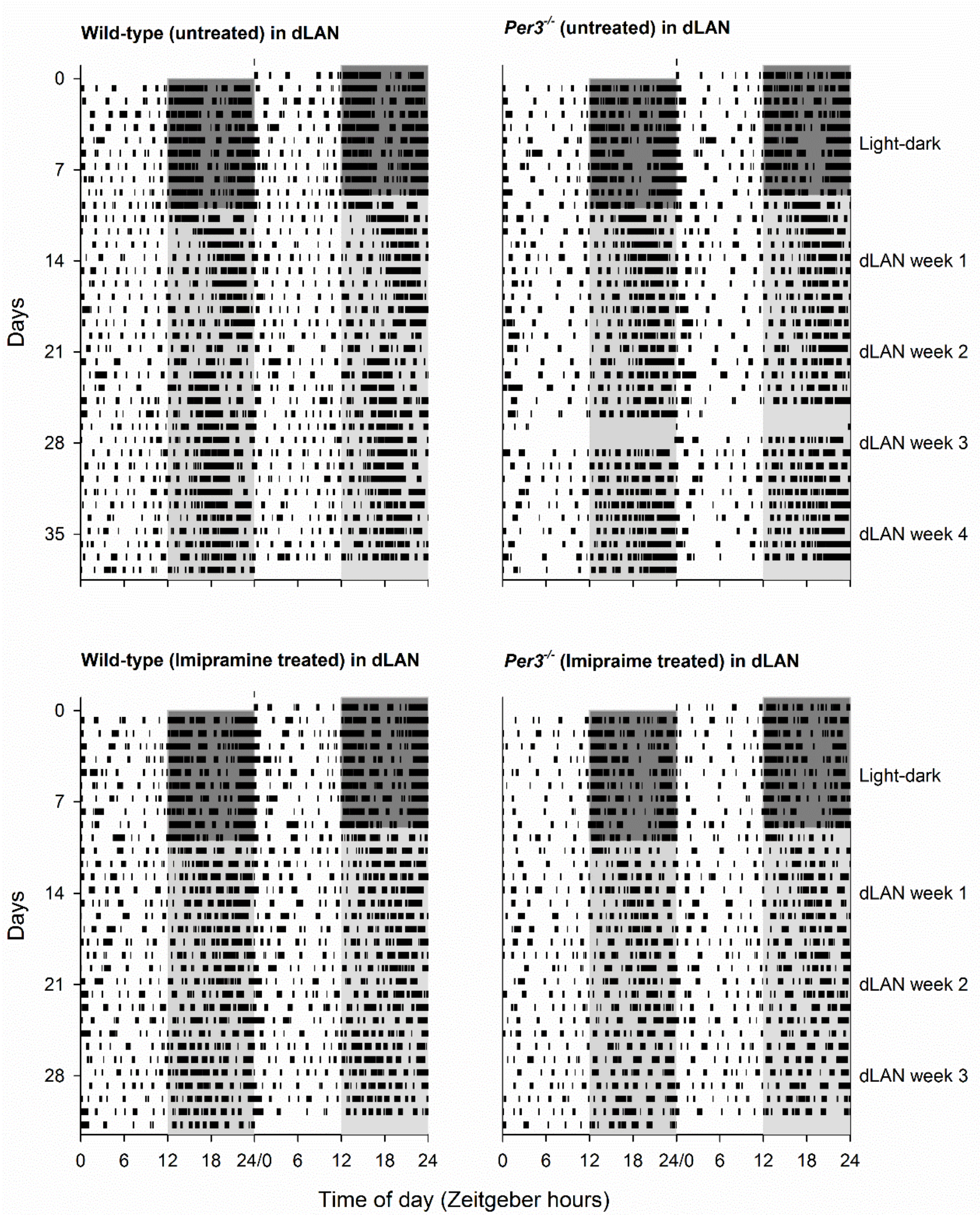

Supplement: Supplemental Figures [file srep40399-s1.pdf]
